# Fructan Structure and Metabolism in Overwintering Plants

**DOI:** 10.3390/plants10050933

**Published:** 2021-05-07

**Authors:** Midori Yoshida

**Affiliations:** NARO Hokkaido National Agricultural Research Center, Sapporo 062-8555, Japan; midori@affrc.go.jp; Tel.: +81-118519141

**Keywords:** fructan, inulin, levan, wintering stresses, fructan exohydrolase

## Abstract

In northern regions, annual and perennial overwintering plants such as wheat and temperate grasses accumulate fructan in vegetative tissues as an energy source. This is necessary for the survival of wintering tissues and degrading fructan for regeneration in spring. Other types of wintering plants, including chicory and asparagus, store fructan as a reserve carbohydrate in their roots during winter for shoot- and spear-sprouting in spring. In this review, fructan metabolism in plants during winter is discussed, with a focus on the fructan-degrading enzyme, fructan exohydrolase (FEH). Plant fructan synthase genes were isolated in the 2000s, and FEH genes have been isolated since the cloning of synthase genes. There are many types of FEH in plants with complex-structured fructan, and these FEHs control various kinds of fructan metabolism in growth and survival by different physiological responses. The results of recent studies on the fructan metabolism of plants in winter have shown that changes in fructan contents in wintering plants that are involved in freezing tolerance and snow mold resistance might be largely controlled by regulation of the expressions of genes for fructan synthesis, whereas fructan degradation by FEHs is related to constant energy consumption for survival during winter and rapid sugar supply for regeneration or sprouting of tissues in spring.

## 1. Introduction

Fructans are oligosaccharides and polysaccharides in which fructose is polymerized with β(2→1) and/or β(2→6) linkage to sucrose [1]. Plants in several families, such as Liliaceae, Asteraceae, Campanulaceae, Boraginaceae, Triticeae, and Asparagaceae, accumulate photosynthetic assimilation products in their vacuoles in the form of fructan rather than starch in the plastid due to strategies for adaptation to their habitat [2,3,4]. Many annual and perennial plants in northern regions accumulate fructan as an energy source that is necessary for the survival of wintering tissues, and degrade fructan for the sprouting or regeneration of tissues in spring. For example, onion, chicory, and asparagus mainly accumulate an inulin type of fructan with β(2→1) linkages in bulbs and roots under the ground for shoot- and spear-sprouting above ground [5,6,7]. In some cereals and temperate grasses in northern regions, fructan is accumulated in all vegetative growth tissues, and the fructan content is associated with freezing tolerance and snow mold resistance of the plant. Fructan in northern monocotyledonous plants is classified as a levan-type fructan, mainly composed of β(2→6) linkages, and it is thought that a levan-type structure is required for adaptation to low-temperature environments [8]. Since the isolation of the 6- sucrose:fructan 6-fructosyltransferase (6-SFT) gene of barley in 1995 [9], fructan synthase genes were isolated from various plants in the 2000s, and the relationships between fructan and environmental stresses such as drought and low temperature have been investigated. Many comprehensive reviews about the issues have been published [10,11,12]. Upstream genes, such as MYB transcription factor, that are related to the activator of gene expression for fructan synthesis have also been analyzed [13,14]. Fructan-degrading enzyme (fructan exohydrolase (FEH)) genes were isolated after the cloning of synthase genes [15]. It has become clear that there are many types of fructan exo-hydrolyzing enzymes in plants with complex-structured fructan, and that they control various kinds of fructan metabolism in growth and survival by different physiological responses. In the first half of this review, the structure and synthesis of fructans are explained. Results of recent studies on fructan synthesis and degradation, with a focus on the roles of FEHs mainly involved in fructan metabolism for field wintering of wheat, timothy, chicory, and asparagus are then introduced.

## 2. Structures and Synthesis of Plant Fructans

### 2.1. Inulin Type of Fructan

#### 2.1.1. Inulin

Inulin is well-known as a human health functional sugar contained in edible parts, such as onion (*Allium cepa*) and garlic (*Allium sativum*) bulbs, in the lily family plants. Inulin is a linear type of fructan in which the fructosyl unit is polymerized by a β(2→1) linkage, and the fructose chain is bound to fructose in sucrose. It is abundantly accumulated in Asteraceae plants such as chicory (*Cichorium intybus*), Jerusalem artichoke (*Helianthus tuberosus*), Datura thistle (*Cynara scolymus*), and the above-mentioned Liliaceae plants [5,16,17,18]. Fructan biosynthesis in most fructan-accumulating plants begins with the synthesis of 1-kestose by sucrose:sucrose 1-fructosyltransferase (1-SST: EC 2.4.1.99). Inulin is synthesized in combination with 1-SST and fructan:fructan 1-fructosyltransferase (1-FFT: EC 2.4.1.100) that polymerizes fructose with β(2→1) linkages [19]. The reaction formulas of these enzymes are as follows (Equations (1) and (2)).

1-SST:

G (glucose)-F(fructose) + G-F → G-F-F (1-kestose) + G(1)

1-FFT:

G-F-F(n) + G-F-F(m) → G-F-F(n − 1) + G-F-F(m + 1) (n (donor) ≥ 1, m (acceptor) ≥ 0)(2)

#### 2.1.2. Inulin Neo Series

There is neo-type inulin in which fructose is β(2→6)-bonded to glucose in sucrose, and a β(2→1)-linked fructosyl unit is also polymerized on the fructose. The inulin neo-series fructans accumulate in bulbs of the lily family, such as onions [20], and the roots of asparagus (*Asparagus spp*.: Asparagaceae) [21]. Fructan:fructan 6^G^-fructosyltransferase (6^G^-FFT: EC 2.4.1.243) catalyzes the first step of transferring fructose to glucose in sucrose/1-kestose. The reaction formulas of the enzyme are shown below (Equations (3) and (4)). 6^G^-FFT also has side activity as 1-FFT [22] and is usually referred to as 6^G^-FFT/1-FFT [7,23]. Regulation of the synthesis and degradation of inulin neo-series in asparagus during winter dormancy and spring sprouting is explained in detail in Section 4.

6^G^-FFT:

G-F + G-F-F (1-kestose) → F-G-F (neokestose) + G-F(3)

G-F-F (1-kestose) + G-F-F (1-kestose) → F-G-F-F(1&6^G^-kestotetraose) + G-F (4)

### 2.2. Levan-Type of Fructan

Levan is a fructan in which fructose polymerized by a β(2→6) linkage is bound to fructose in sucrose. It is produced by Triticeae plants, such as wheat (*Triticum aestivum*) and barley (*Hordeum vulgare*), and by temperate grasses such as orchardgrass (*Dactylis glomerata*) and perennial ryegrass (*Lolium perenne*) [8,24]. Timothy (*Phleum pratense*) and orchardgrass accumulate linear chain levan. Many plants synthesizing levan have β(2→1)-linked fructose side chains and residues with a main levan frame, and accumulate various complex-branched fructans in tissues at the same time [25,26]. These fructans are generically called levan-type fructans.

#### 2.2.1. Graminan

Graminan, which is a complex branch of fructose in β(2→1) and β(2→6) linkages to sucrose, accumulates in vegetative tissues such as the foliage and roots of Triticeae plants, including wheat and barley [25,27,28]. The key enzyme, 6-SFT, plays an important role in the synthesis of levan-type fructans, including graminan in combination with 1-SST and 1-FFT. 6-SFT forms and extends β(2→6)-linked fructose in fructans [29]. The reaction formula of 6-SFT is as follows (Equation (5)):

6-SFT:

G-F + G-F-F(m) → G + G-F-F(m + 1) (m ≥ 1)(5)

**Figure 1:**
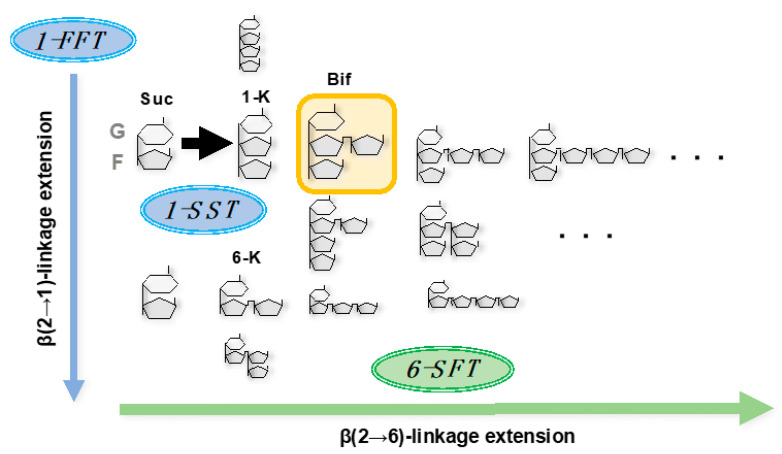
Illustration of fructan synthesis in wheat. G, glucose; F, fructose; Suc, sucrose; 1-K, 1-kestose; 6-K, 6-kestose; Bif, bifurcose.

Graminan is formed in a process in which fructose is polymerized around the base saccharide, bifurcose (1&6-kestotetraose), which is formed with β(2→6)-linked fructose attached to 1-kestose (Figure 1). Since the isolation of the barley 6-SFT gene [9], fructan synthase gene isolation and clarification of its enzymatic properties have been actively reported. Our group [30,31] first isolated genes of all types of wheat fructosyltransferase (1-SST: *wft2*, AB029888; 6-SFT: *wft1*, AB029887; 1-FFT: *wft3* and *wft4*, AB088409 and AB088410, respectively). In our study, using recombinant proteins produced by *Pichia pastoris* induced with a cloned gene, we revealed that wheat 6-SFT prefers the fructans of 1-kestose, and β(2→6)-linked fructose extended to 1-kestose rather than 6-kestose and β(2→6) linkage levan as acceptors. In addition, we discovered that wheat 1-FFT also prefers the levan and fructans with β(2→6)-linked fructose extended to 1-kestose rather than β(2→1)-linkage inulin as acceptors. These characteristics of the enzymes are involved in the construction of the complex branched structure of graminan [31].

#### 2.2.2. Levan Neo Series

There is a levan-type fructan that has a structure in which fructosyl units are polymerized by a β(2→6) linkage to glucose in sucrose, and it is called a levan neo series. 6^G^-FFT catalyzes the first step of transferring a fructose to glucose, as in the synthesis of the inulin neo series. Very complex branched fructans with a wide variety of structures are accumulated in perennial ryegrass, in which synthesis of levan neo series has been investigated in detail [32,33]. 6-SFT is responsible for the transition of fructose with a β(2→6) linkage in levan neo series. In perennial ryegrass, 1-kestose is synthesized by 1-SST, but unlike the graminan-type plants described in Section 3.1, bifurcose (tetrasaccharide) is hardly accumulated, or its amount is extremely small. From analysis using recombinant proteins of the 6-SFT gene (*Lp6-SFT*) and the 6^G^-FFT gene (*Lp6^G^-FFT*) of perennial ryegrass, the reason for this is thought to be that synthesis of neokestose (trisaccharide) or 1&6^G^-nystose (1&6^G^-kestotetraose:tetrasaccharide) by 1&6^G^ -FFT is the dominant reaction for 1-kestose as a substrate. This means that 6^G^-FFT has a higher affinity for 1-kestose than 6-SFT in perennial ryegrass. The presence of this 6^G^-FFT and its high affinity for 1-kestose may construct the structure of the levan neo series in perennial ryegrass [34]. Neo-type fructans were detected in wheat flour and it was revealed that neo-levan and neo-type inulin oligosaccharides accumulated during the development of wheat seeds [35]. It has been shown that 6^G^-FFT might function in wheat during the seed development period. It is thought that the accumulation of fructans with various structures might be advantageous as a physiological mechanism of the tissues at each growth stage of plants.

#### 2.2.3. Linear Levan

Although many levan-type fructan-synthesizing plants accumulate a large amount of branched-type fructans, timothy, a temperate grass, produces a highly polymerized linear levan that has been reported to reach about 90 degrees of polymerization (DP) in vegetative tissues [8,36]. Tamura et al. [37,38] isolated two 6-SFT genes (*PpFT1*, AB436697; *PpFT2*, AB822634) with different enzymatic properties. Timothy 6-SFT has a lower affinity for sucrose than that of other plants, and fructans are therefore not synthesized unless the concentration of sucrose is high. A comparison of the enzymatic properties of the two genes, *PpFT1* and *PpFT2*, using recombinant proteins synthesized in *P. pastoris* showed that the *PpFT1* protein synthesizes levan with an extremely high degree of polymerization under the condition where sucrose is continuously supplied. The *PpFT1* protein can also synthesize 1-kestose, and fructans with β(2→1)-linked fructose side chains were also detected by HPLC analysis in the synthesized high DP levan. The recombinant PpFT2 protein synthesized 6-kestose and short β(2→6)-linked levans up to about DP 6 under an incubation condition of 1 M sucrose for 96 h. Furthermore, as an attempt to produce levan in a transgenic plant, we introduced these two genes into sugar beet (*Beta vulgaris*), and succeeded in producing sugar beet in which most of the sucrose that accumulated in the roots was converted into fructan [39]. Analysis of the accumulated fructans in the beet roots of the transformants revealed that *PpFT1*-introduced sugar beet accumulated the same highly polymerized levan as that produced by the *P. pastoris* recombinant protein mentioned above at a high concentration. Conversely, in the roots of sugar beet in which *PpFT2* was introduced, levan with a linear β(2→6) linkage of about DP 3–40 accumulated at a high concentration. It has been shown that timothy *PpFT1* prefers fructans with a higher degree of polymerization over wheat 6-SFT as a substrate. These results indicate that high DP linear levan in timothy is produced by *PpFT1* using the β(2→6)-linked levan synthesized by PpFT2 as a substrate.

## 3. Plant Fructan and Freezing Tolerance

### 3.1. Physical Roles of Fructan in Drought and Freezing Tolerance

It is well known that wintering plants increase their sugar content in their tissues when exposed to low temperatures (≥0 °C) during cold acclimation (see Section 3.2). These plants also increase freezing tolerance during cold acclimation [8,11,12]. It was reported that fructan content in tissues was correlated with the freezing tolerance of plants [40]. Degrees of polymerization of the fructan that plants produce are from oligosaccharides to DP × 10^2^ [36]. Plant fructans with that DP range are thought to be highly hydrophilic, and this characteristic of affinity to water molecules is closely related to tolerance for drought. Some of the northern cereals and temperate grasses that originated in drought areas in the highlands of Southwest Asia store photosynthetic assimilation products as water-soluble fructans in vacuoles, not as insoluble starch in plastids of vegetative tissues. It is thought that fructans in vacuoles might regulate the osmotic pressure of cells as a rational strategy for drought resistance. The ability of inulin to protect the cell membrane against dehydration has been proven in in vitro studies [10,11]. In addition, the radical scavenging ability of fructan was proposed to be similar to that of the raffinose oligosaccharide family [41,42]. These multiple factors may result in a freezing tolerance through which plant cells withstand dehydration due to extracellular freezing in tissues at low temperatures below 0 °C.

### 3.2. Fructan Accumulation during Cold Acclimation

In northern regions, winter cereals and temperate grasses not only suffer from severe winter conditions, such as freezing and persistent snow cover, but also from infection by snow molds and psychrophilic fungi such as *Typhula ishikariensis* and *Microdochium nivale* under snow [43,44]. It was reported that snow mold resistance is strongly associated with the fructan content in plants [40]. It was previously reported that sugar contents and expression of fructan synthesis genes such as *1-SST*, *6-SFT* and *6^G^-FFT* increase in wheat, timothy, and perennial ryegrass during cold acclimation [30,33,38]. Regarding synthesis of fructans during that period, please refer to reviews [8,12]. Since fructan is a polymer, fructan may not have an influence on excessive increase in osmotic pressure, and the amount of accumulated fructan reaches more than 10% of fresh weight in some plants in early winter. Sucrose is translocated from photosynthetic organs to sugar-storage organs, which differ among plants. In addition, fructan structures are varied, with complex forms. A large amount of fructan plays a main role as an energy storage substance for the survival of tissues in winter and the regeneration or sprouting of tissues in spring.

## 4. Fructan Degradation in Wintering Plants

### 4.1. Fructan Exohydrolase

Fructan, a final product of metabolism, is degraded by FEHs that hydrolyze the fructosyl unit at the end of fructan to release fructose. Fructosyl units with β(2→1) linkage and β(2→6) linkage are hydrolyzed by 1-FEH (EC 3.2.1.153) and 6-FEH (EC 3.2.1. 154), respectively. The reaction formula of an FEH is shown below (Equation (6)). Recently, it has been clarified that there are many types of FEH enzymes with different substrate specificities in plants [15,45]. In this section, the roles of various FEHs in degradation of fructan in wintering tissues of wheat, timothy, asparagus, and chicory are described.

FEH:

G-F-Fn → G-F-F (n − 1) + F (n ≥ 1)(6)

### 4.2. Gramineae

#### 4.2.1. Wheat Fructan Exohydrolase

**Figure 2:**
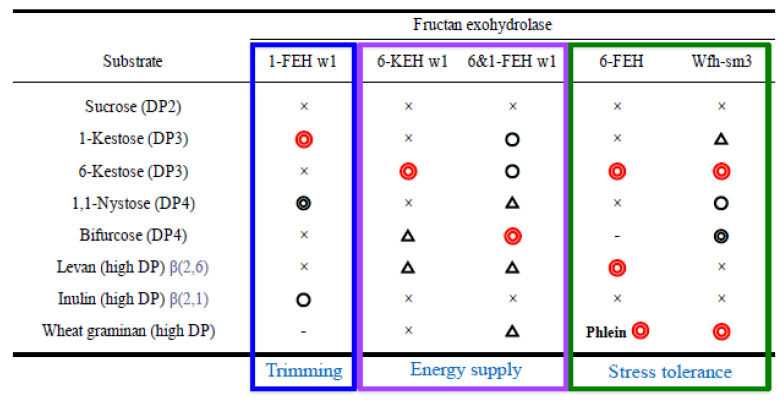
Substrate specificities of wheat FEHs and their supposed roles. Symbols show the degradation levels with recombinant enzymes produced by *Pichia pastoris*: Red double circle, most highly hydrolyzed; black double circle, highly hydrolyzed; circle, hydrolyzed; triangle, weakly hydrolyzed; cross, hardly hydrolyzed; not examined. References of genes: *1-FEHw1* [46], *6-KEHw1* [50], *6&1-FEH* w1 [49], *6-FEH* [46], and *Wfh-sm3* [51].

Wheat accumulates graminan (fructan) in vegetative tissues in autumn, and stores a large amount of fructan in crown tissues for the long winter. Several genes of wheat FEHs have been isolated [12,28]. Functional analysis of the *P. pastoris* recombinant proteins of these clones revealed that there are multiple genes and homologs with different substrate specificities because of the complex structures of graminans (Figure 2). These genes are enzymatically classified into five kinds of wheat FEH: 1-FEH (*1-FEH w1*, *w2* and *w3*, AJ516025, AJ508387 and AJ564996), 6-FEH (*6-FEH*, AM075205), 6-KEH (*6-KEH w1 and w2*, AB 089271 and AB089270), 61-FEH (*6&1-FEH w1*, AB089269) and Wfh-sm3 (*wfh-sm3*, AB196524) [46,47,48,49,50,51]. Among these wheat FEHs, Wfh-sm3, a 6-FEH with side activity of 1-FEH, is the only enzyme that is able to hydrolyze all components of wheat graminan [51].

#### 4.2.2. Role of FEH in Wintering of Wheat

Fructan level is the highest among carbohydrates in winter wheat tissues just before snow cover, and decreases under snow until snow melt, when photosynthesis restarts. The remaining level of fructan at the end of snow cover is strongly related to snow mold resistance [40,44]. Fructan is metabolized by FEHs during both the seasons for accumulation and consumption. It has been proven that freezing tolerance and snow mold resistance of wheat increase during the process of cold acclimation and decrease under snow cover [40,43]. These changes are associated with fructan content controlled in a balance between the expression of genes for synthesis and degradation. Namely, regulation of the expression of various FEH genes plays a crucial role in the overwintering ability of wheat. We reported the results of qRT-PCR analyses of changes in expression levels of fructan synthase and hydrolase genes in leaves and crowns from autumn to spring [52]. Transcript levels of the wheat 1-SST gene increased during cold acclimation in autumn, and then decreased with ambient sub-zero temperatures, and continued to be expressed at low levels under snow. Transcript levels of 1-SST in leaves of a snow-mold-resistant cultivar that accumulates a large amount of fructan are remarkably high throughout the winter. It was suggested that wheat 1-FEH may play a role in trimming of the β(2→1)-linked fructosyl unit during fructan synthesis [46]. Among wheat FEHs, changes in the expression of *wfh-sm3* corresponded to changes in and levels of fructan content in wheat cultivars. The expression of *wfh-sm3* in crowns and leaves of wheat cultivars was repressed during a freezing period when minimum temperatures were sub-zero before snow cover. The transcript levels of the gene increased under snow and then decreased toward the end of snow cover [52]. It was also shown that the transcript levels of *wfh-sm3* in crown tissues of snow-mold-resistant cultivars with high levels of fructan were lower than those in other wheat cultivars. The *wfh-sm3* gene was isolated from snow-mold-inoculated (*T. ishikariensis*) wheat leaves. It was also reported that expression of *wfh-sm3* was induced in wheat leaves with snow mold under snow cover, being consistent with the rapid decrease in fructan content in the tissues [51]. *Wfh-sm3* plays a key role in the regulation of both seasonal changes and varietal differences in fructan metabolism for wintering survival of wheat. In wheat exposed to severe freezing before snow cover, the contents of mono- and disaccharides are predominantly associated with freezing tolerance [40]. Expression of the wheat 6-FEH gene was induced in wheat tissues in the freezing season. Wheat 6-FEH is able to hydrolyze the β(2→6)-linked fructosyl unit from 6-kestose to high DP β(2→6)-linked fructans (phlein) [48]. The expression of this gene may be related to an increase in the freezing tolerance of wheat. The changes in transcript level of 6&1-FEH were rather constant during autumn and winter [52]. After snowmelt, wheat plants rapidly regenerated using sugar stored in crowns. The transcript levels of 6-KEH in all cultivars were repressed under snow cover and then suddenly increased after snowmelt [52]. The gene may be related to spring growth of wheat. Conversely, it was suggested that the wheat 6-KEH enzyme might be localized in apoplastic spaces of wheat tissues [50]. The existence of a FEH in apoplasts against freezing, pathogens, or microbes has also been proposed in other plants [53,54,55]. The results of those studies suggest that there are some FEH genes in wheat and that they have various roles in cold accumulation and wintering (Figure 2).

#### 4.2.3. Timothy FEHs and Their Role in Wintering

As mentioned in Section 2.2.3, DP of accumulated fructan in timothy during cold acclimation is higher than those of other temperate grasses, such as perennial ryegrass and meadow fescue [8]. The structure is long linear levan. A 6-FEH gene of timothy, *Pp6-FEH1* (AB583555), was cloned [56]. The recombinant enzyme of *Pp6-FEH1* shows hydrolysis activity to β(2→6)-linked fructosyl units and degraded substrates from oligo to long linear levan. Seasonal changes in gene expression of *PpFT1* and *PpFT2*, both coding 6-SFT, and *Pp6-FEH1* and enzymatic activities of FT and 6-FEH in the crowns of timothy seedlings grown in Sapporo from autumn to spring were reported [38]. The transcript levels of *Pp6-FEH1* slowly increased under snow, but 6-FEH activity in the crown seemed to be constant during the season. However, the transcript levels of *PpFT1* and *PpFT2* in autumn were high and they repressed in low levels during the snow cover season. The changes corresponded to those of FT activity. These results suggested that fructan content in timothy crown tissue is regulated by fructan synthesis, whereas timothy FEH may play a role in sugar supply for energy consumption in the low temperature season. Similar to wheat *wfh-sm3*, the expression of *Pp6-FEH1* was strongly induced in crown tissues inoculated with snow mold (*T. ishikariensis*) and the contents of fructan in the tissues remarkably decreased [38].

### 4.3. Asparagaceae

#### Fructan Metabolism in Asparagus Roots during Winter and Spring

Asparagus (*Asparagus officinalis*), a perennial plant, is cultivated as an edible crop in areas with various climates. It is also grown in northern regions with freezing temperatures in winter. Asparagus accumulates an inulin type of fructan in root tissues [57]. The roots survive under the ground during winter. In spring, spears sprout using sugar released from the stored fructan in roots. Ueno et al. [58] isolated a novel *6G&1-FEH* gene (*aoeh4a*, LC314428; *aoeh4b*, LC314429) from asparagus. The recombinant enzyme hydrolyzed neokestose into sucrose and fructose and hydrolyzed β(2→1)-linked fructosyl units of nystose and fructosylnystose, but minimally reacted to 1-kestose. They analyzed the expression of *6G&1-FEH* and fructosyltransferase genes in asparagus roots using rootstock-planting forcing culture, in which field grown asparagus roots dug out in mid-autumn were stored at 2 °C for 3 weeks to break dormancy and then transplanted in a greenhouse for the harvest of sprouting spears of asparagus [58,59]. During low-temperature preservation, the transcript of *6G&1-FEH* remarkably increased in roots, whereas fructan content remained at a high level, since the expression levels of fructosyltransferase genes, 6^G^-FFT (*aoft1*, AB084283), 1-FFT (*aoft2*, AB115554), and 1-SST (*aoft3*, AB115555), were also high in the same period. Conversely, the transcript of *6G&1-FEH* gradually increased corresponding to the decrease in fructan content in roots after transplanting in a greenhouse. The released sugar may be used for spear sprouting. In this harvest period, the expression of all of the *6G-FFT*, *1-FFT* and *1-SST* genes is repressed in roots. Similarly, coordinate regulation of the expression of genes between fructan synthesis and degradation in roots must be carried out in field wintering asparagus. Although not discussed in this review because the plant is beyond the scope of this Special Issue, agave (*Agave spp*.), a tropical habitat plant also in the family of Asparagaceae, synthesizes fructan. Agave fructan, which is well-known as a fermentation material for tequila, is very complex and there are all forms of fructan in agave because of the survival of plants in a severe drought environment. Investigation of the enzymes present in agave fructan metabolism has advanced [60,61].

### 4.4. Asteraceae

#### Regulation of FEH Gene Expression in Chicory Taproots in Winter

Chicory is cultivated as the most important crop for commercial production of inulin, which is largely accumulated in taproots. Native chicory is a perennial plant that can survive severe freezing and snow cover environments [62]. Among plant FEHs, chicory root FEH genes were cloned first (*1-FEH I*, AJ242538; *1-FEH IIa*, AJ295033; *1-FEH IIb*, AJ295033) [63,64]. In field-grown chicory, the contents of inulin in taproots increase during summer growth, and large amounts of inulin are maintained in winter. It was reported that the increase in inulin content depended on the increase in fructosyl polymerization, and that the highest DP was observed in September [65]. It is thought that the phase change in fructan metabolism in chicory taproots might occur in early October [65,66]. During the changes in inulin content and DP status, the gene expression level of 1-SST and enzyme activity level in taproots are high in early summer and then linearly decrease from summer to winter, whereas those of 1-FFT are constant. Conversely, gene expression levels of 1-FEH I and 1-FEH II and FEH activity levels are low until October and then increase after October [65,66]. These findings indicate that the expression of chicory FEH genes is induced by low temperatures in the field. Cold-induced expression of FEH genes was also detected in asparagus roots stored at 2 °C [58]. This cold regulation of chicory FEH genes was investigated by expression analysis of promoter-reporter genes, and stress-responsive *cis*-elements were found in the promoter [67]. Recently, regulation of the expression of chicory FEH genes by R2R3-MYB transcription factors (CiMYB5 and CiMYB3) was proved using a hairy root culture [68]. The R2R3-MYB transcription factor is thought to regulate the expression of genes for fructan synthesis in wheat [13]. The roles of fructan metabolism in response to cold in roots as storage tissues may be different from those in vegetative tissues, such as leaves and stems.

## 5. Conclusions

The results of studies of fructan metabolism in plants during winter that were described here show that changes in fructan contents in wintering plants associated with freezing tolerance and snow mold resistance might be largely controlled by regulation of the expression of genes for fructan synthesis, whereas fructan degradation by FEHs is related to constant energy consumption for survival during winter and rapid sugar supply for regeneration or sprouting of tissues in spring. There are FEHs such as wheat Wfh-sm3 and 6-FEH, for which gene expression might regulate changes in contents of fructan and mono- and disaccharides associated with winter stresses. Since the roles of FEHs in plants vary, different pathways may be involved in FEH genes. Further investigation of the regulation of FEHs will be a difficult task, but it is necessary for understanding the strategies of fructan-accumulating plants for adaptation to northern habitats.
